# Alleviation of soil acidification and modification of soil bacterial community by biochar derived from water hyacinth *Eichhornia crassipes*

**DOI:** 10.1038/s41598-023-27557-9

**Published:** 2023-01-09

**Authors:** Rumpa Jutakanoke, Nuttakorn Intaravicha, Purin Charoensuksai, Wuttichai Mhuantong, Jarungwit Boonnorat, Jirapast Sichaem, Wongsakorn Phongsopitanun, Warunya Chakritbudsabong, Sasitorn Rungarunlert

**Affiliations:** 1 Department of Microbiology and Parasitology, Faculty of Medical Science, Naresuan University, Mueang, Phitsanulok, 65000 Thailand; 2 Faculty of Medical Science, Centre of Excellence in Medical Biotechnology (CEMB), Naresuan University, Phitsanulok, 65000 Thailand; 3 Environmental Science and Technology Program, Faculty of EnvironmentalScience and Technology, Pathumwan Institute of Technology, Bangkok, 10330 Thailand; 4 Department of Biopharmacy and Bioactives from Natural Resources Research Collaboration for Excellence in Pharmaceutical Sciences, Faculty of Pharmacy, Silpakorn University, Nakhon Pathom, 73000 Thailand; 5 National Center for Genetic Engineering and Biotechnology (BIOTEC), National Science and Technology Development Agency (NSTDA), Pathum Thani, 12120 Thailand; 6 Department of Environmental Engineering, Faculty of Engineering, Rajamangala University of Technology Thanyaburi (RMUTT), Klong 6, Pathum Thani, 12110 Thailand; 7 Research Unit in Natural Products Chemistry and Bioactivities, Faculty of Science and Technology, Thammasat University Lampang Campus, Lampang, 52190 Thailand; 8 Department of Biochemistry and Microbiology, Faculty of Pharmaceutical Sciences, Chulalongkorn University, Bangkok, 10240 Thailand; 9 Laboratory of Cellular Biomedicine and Veterinary Medicine, Faculty of Veterinary Science, Mahidol University, Nakhon Pathom, 73170 Thailand; 10 Department of Preclinic and Applied Animal Science, Faculty of Veterinary Science, Mahidol University, Nakhon Pathom, 73170 Thailand

**Keywords:** Soil microbiology, Microbiology, Environmental microbiology

## Abstract

The highly acid sulfate Rangsit soil series of Rangsit, Pathum-Thani district, Thailand poses a major problem for agriculture in the area. Water hyacinth is a naturally occurring weed that can grow aggressively, causing eutrophication and leading to many severe environmental impacts. Here, through the pyrolysis process, we convert water hyacinth to biochar and use it for acid soil amendment. We found the ratio between biochar, soil, and sand suitable for the cultivation of water convolvulus to be 50 g of biochar, 400 g of soil, and 100 g of sand (1:8:2). This soil mixture improved the pH of the soil from 4.73 to 7.57. The plant height of the water convolvulus grown in the soil mixture was the greatest at 20.45 cm and the plant weight with and without roots was greatest at 2.23 g and 2.52 g, respectively. Moreover, we demonstrated the dominance and high abundance of *Bacillus* among the community in soil with biochar amendment. Here we provide the first assessment of the appropriate amount of water hyacinth-derived biochar for mitigation of soil acidity and promotion of optimal water convolvulus growth. Moreover, biochar can optimally modify soil bacterial communities that benefit plant development.

## Introduction

Acid sulfate soil is the primary agricultural problem in Rangsit, Pathum-Thani District, Thailand. A former alluvial plain, the area developed when freshwater from the Chao Phraya River flowed into a former tidal flat, triggering an accumulation of marine sediments and riverine alluvium under brackish water conditions^1^. The accumulation of organic matter with microorganisms under anaerobic conditions leads to the formation of Fe^3+^ and SO^2−^_4_ which, under certain circumstances, forms pyrite (FeS_2_)^2,3^. When the soil is drained, the pyrite, exposed to oxygen, is oxidized into sulfuric acid^2,3^, rendering it extremely acidic (pH 4.0–5.0) and harmful to plants and the environment. Moreover, in an intensely acidic condition, soil will dissolve manganese, causing toxicity in the soil. It is therefore necessary to find methods to alleviate soil acidity.

Water hyacinth (*Eichhornia crassipes*) is a notorious invasive aquatic weed^4^. As the plant consumes high levels of nitrogen and phosphorus in bodies of water, it grows out of control, causing water pollution and negatively impacting transportation and human health^5,6^. The water hyacinth can produce a high biomass yield (100–120 t ha^–1^ year^–1^) containing essential plant nutrients and a valuable cellulose content that is essential for soil reclamation^7^. Water hyacinth-derived biochar can support carbon stability and enhance long-term carbon storage in the soil as the cellulosic carbons in the water hyacinth are converted to aromatic carbon—the stable form of carbon in biochar—through the pyrolysis process^8^. This strategy of converting recalcitrant weed to useful biochar—thanks to its soil amendment and carbon sequestration properties—is valuable in both weed management and soil improvement^7,8^.

Biochar amendment can alter bacterial communities in soil by changing soil pH, cation exchange capacities, organic matter, C/N ratio, water holding capacity, and availability of nutrients^9–11^. 16s rRNA sequencing reveals that such effects on the soil lead to increased microbial diversity and microbial community modification^10^. The alteration of the microbiome in soil causes changes in the metabolic activity of microorganisms, thus influencing plant nutrient uptake and crop productivity^12,13^. Wang et al.^10^, reported that biochar application can alleviate negative plant–soil feedback through changes in the soil microbiome. Several studies have demonstrated the promotion of plant resilience and protection against soilborne pathogen invasion conferred by high richness and diversity of soil microbes^14,15^.

Some studies report the beneficial use of biochar produced from water hyacinth for soil amendment. Masto et al.^16^, reported significant increases in pH in red soil with a neutral pH after application of biochar produced from the plant. The study also showed that the addition of biochar to red soil can improve maize growth (germination and shoot and root weight)^16^. Najmudeen et al.^17^, demonstrated that 4% of water hyacinth-derived biochar in the soil mixture causes the highest growth in pokkali compared with the control, and that biochar also decreases the pH of water in the *pokkali* paddy-fish culture system.

Dry and crushed biomass of *E. crassipes* can be used to develop a biotreatment for the adsorption of Chromium from contaminated water^18^. Sayago et al.^18^, found the diffusion speed (kf) and adsorption capacities (Ks) of the biomass of *E. crassipes*. These values can be applied to the design of the system of treatment to improve the Chromium adsorption capacities^18^. Sayago et al.^19^, reported that *E. crassipes* can totally be used to eliminate the Chromium from water and used the biomass contaminated with Chromium to produce bioethanol. However, no other report regarding acid soil amelioration by water hyacinth-derived biochar exists.

Here, we firstly used water hyacinth-derived biochar for acid soil amendment to the Rangsit soil series and investigated the bacterial community in the amended soil. We converted water hyacinth to biochar through the pyrolysis process and varied the ratio between biochar and soil to determine the optimal growth-promoting ratio for water convolvulus. We also determined the quality of the soil mixture after soil amelioration. We performed the microbiome study by high-throughput 16S amplicon sequencing to analyze the changes in the bacterial community in the soil mixture alleviated using water hyacinth-derived biochar.

## Methods

### Preliminary experiment

#### Biochar preparation and characterization

The water hyacinth biomass was taken from Pornthisarn canal, Rangsit, Pathum-Thani district, Thailand (14°02′01.0′′ N 100°43′56.0′′ E). A voucher specimen (*Eichhornia crassipes* (Mart.) Solms) was identified by Asst. Prof. Dr. Pranee Nangnga, a taxonomist or botanist, and deposited in the herbarium of the Department of Biology, Faculty of Science, Naresuan University, Thailand. The voucher specimen was PNU 5844. *Eichhornia crassipes* (Mart.) Solms is not endangered. The specimen was collected in the field with permission from the Department of Agriculture of Thailand. The study of this species complied with relevant institutional and national guidelines and legislation. After collecting plant trunks and leaves we air dried them at a temperature of 37 ℃ for 5 days. We performed pyrolysis on 10 kg of dried water hyacinth biomass by heating at a temperature of 500 ℃ for 1 h under a limited O_2_ supply condition adapted from the pyrolysis methods of Masto et al.^16^. We then finely ground up 10 g of obtained biochar and added it to a 250 ml plastic bottle containing 50 ml deionized water. We shook the mixture by hand for 1 min and allowed it to stand for 24 h before measuring pH and electroconductivity (EC). We determined the biochar particle size by passing the particles through a 0.075 mm sieve. We also measured cation exchange capacity (CEC) using the method of Zhang et al.^20^.

#### Rangsit soil series sampling and chemical property analysis

We collected 30 kg per soil sample from farmlands of Klong 4, 5, and 6 of Rangsit, Pathum-Thani district, Thailand at a depth of 30 cm from the soil surface (Supplementary Fig. 1). We dried the soil samples in an indoor area, ground them, and sifted them through a 2 mm sieve. We subsequently analyzed the soil samples for pH, EC, CEC, and organic matter (OM)—phosphorus (P), exchangeable calcium (Ca), magnesium (Mg), and potassium (K). We performed Phosphorus determination by using Bray II extractant to extract available phosphorus from the soil samples and subsequently filtered the extracted Phosphorus using Whatman qualitative filter paper No. 5. The filtrate was developed with Molybdenum blue and measured absorbance with spectrophotometer at 820 nm. For Ca, K and Mg determination, we extracted the soil samples with 1 N NH_3_OAc and used the solution that we obtained from the extraction for Ca, K and Mg determination by using Atomic absorption spectroscopy^21^.

#### Determination of appropriate ratio of biochar to soil sample for cultivation

We assigned a 3 × 4 factorial in a completely randomized design (CRD) to this experiment. The first variable, with three levels, represented the three places we collected the soil samples, as follows: Klong 4, Klong 5, and Klong 6. The second variable, which has four levels, represented the ratio between biochar and soil sample, as follows:

- 0 g of biochar to 10 g of soil sample (control group).
- 0.25 g of biochar to10 g of soil sample.
- 0.5 g of biochar to 10 g of soil sample.
- 1.0 g of biochar to 10 g of soil sample.

We first sifted the soil through 2 mm sieves and then mixed it with the biochar according to the soil–biochar ratios defined above.

We mixed the soil–biochar mixture with 50 ml deionized water in a 250 ml plastic bottle, whereafter we shook the suspensions for 1 min and allowed them to stand for 24 h before measuring pH and EC^21^. We performed the measurement in 4 replicates.

### Main experiment

#### Analysis of growth of water convolvulus and the soil mixture after cultivation with biochar-treated soil

We assigned a 3 × 3 factorial design in a CRD to this experiment. The first variable, with three levels, represented the three locations where we collected the soil samples, as follows: Klong 4, Klong 5, and Klong 6. The second variable had three levels representing the ratios between biochar, soil sample, and sand. Since the soil samples we collected were clay soil unsuitable for plant and crop cultivation, we mixed the soil with sand to improve the soil texture from clay soil to clay loam soil, with the following ratios:

- 0 g of biochar, 400 g of soil, and 100 g of sand (control group).
- 25 g of biochar, 400 g of the soil, and 100 g of sand.
- 25 g of biochar, 400 g of the soil, and 100 g of sand.
- 50 g of biochar, 400 g of the soil, and100 g of sand.

We thoroughly mixed the components in the control and each treatment group and then put 500 g of each in the planting bags. We randomly arranged the planting bags of each treatment group and watered them for 30 days. We subsequently grew the water convolvulus by putting 5 seeds of the plant, previously soaked in water for 24 h, in each of the previously prepared planting bags, and cultivated them outdoors. We watered the plants twice a day (morning and evening). We measured the shoot height of the plants with a ruler at 7, 14, and 21 days. After 23 days of cultivation, we harvested the water convolvulus. We evaluated the plant growth on the last day of cultivation by measuring the fresh weight of the water convolvulus with and without roots and the shoot height.

To examine the chemical properties of the soil mixtures after cultivation, we collected the cultivated soil mixtures and dried them indoors for 7 days, thereafter grinding the soil mixtures and sieving them through 2 mm sieves and determining the pH and EC of each mixture, respectively^21^.

#### Statistical analysis

We performed two-way analysis of variance to analyze the pH and EC of the soil mixtures and weight and height of the plant shoots to compare the means of different treatments. We obtained least significant differences at P < 0.05 using Duncan's multiple range test (DMRT). We used SPSS statistics-version 23.0^22^ for our statistical analysis.

### Microbiome in the soil

#### 16S Amplicon sequencing

We divided the samples into two groups: (1) 0 g of biochar, 400 g of soil, and 100 g of sand as a control, and (2) The optimal condition for the growth of water convolvulus under the condition of our studies. We processed them for metagenomics DNA extraction using the QIAamp PowerFecal Pro DNA Kit. We performed the first stage polymerase chain reaction (PCR) to amplify the 16S rRNA sequences from a DNA sample using a region of interest-specific primers targeting the V3-V4 variable region. We added microbial DNA, 16S Amplicon PCR forward primer, 16S rRNA Amplicon PCR reverse primer, and 2 × KAPA HiFi Hotstart Ready Mix to a 96-well 0.2 ml PCR plate to initiate the PCR. We sealed the plate and performed the PCR in a thermocycler. We started with the degeneration stage, using 95 ℃ for 3 min, followed by the annealing stage, using 25 cycles of 95 ℃ for 30 s, 55 ℃ for 30 s, and 72 ℃ for 30 s, respectively, and finally ended with the extending stage, using 72 ℃ for 5 min. We ran the 1 µL PCR product on a Bioanalyzer to verify the size. Our expected size for the Bioanalyzer trace following the Amplicon PCR step was approximately 550 base pairs. Thereafter, we used Ampure XP beads to purify the V3 and V4 amplicon away from free primers and primer–dimer species. Thereafter, we performed the second stage PCR for attaching the dual indices and Illumina sequencing adapters using the Nextera XT Index Kit, after which we used the Ampure XP beads to clean up the final library before quantification. To validate the size, we ran 1 µL of a 1:50 dilution of the final library on a Bioanalyzer DNA 1000 chip. We expected a size on a Bioanalyzer trace of the final library of approximately 630 base pairs. Before pooling, we quantified and normalized the library using a fluorometric quantification method. Before loading it into the MiSeq reagent cartridge, we denatured the pooled library with NaOH, diluted with hybridization buffer, and heated it. We sequenced the samples on MiSeq using paired 300-bp reads and MiSeq v3 reagent kits for improved run metrics. We overlapped the ends of each read to obtain high-quality, full-length readings of the V3 and V4 regions of the microbial 16S rRNA gene. The MiSeq run output can generate over 100,000 reads per sample. We constructed the genus-level classification demonstrated in the taxonomic plot by analyzing the MiSeq sequencing data using the MiSeq Reporter software. We conducted all the above-mentioned procedures at the OMICS Science and Bioinformatics Center, Chulalongkorn University.

#### Data analysis

We initially performed the quality control of raw pair-end (PE) reads by removing low-quality sequences (Q score < 20) and ambiguous bases (N base) using FASTP^23^. We based the whole analysis workflow on QIIME2 version 2020.6^24^ We merged, de-noised, and clustered cleaned PE sequences to the amplicon sequence variants (ASVs) via dada2^25^ implemented in QIIME2. To avoid biases from imbalances in sampling depth in comparison of samples, we rarefied the ASV table to an even depth of 10,000 sequences per sample, which corresponded to the number of sequences present in the sample with the lowest number of sequences. After sub-sampling, we computed the alpha diversity index (Chao1 and Shannon indices) by the core-metrics-phylogenetic QIIME2 diversity plugin. We calculated the Bray–Curtis dissimilarity matrix for measuring the differences among bacterial communities and visualized it via principal coordinate analysis (PCoA). We performed the taxonomic classification using the “classify-consensus-blast” method against the Silva database^26^. We statistically analyzed and plotted the bacterial profile and diversity index using STAMP^27^ and the “plotly” package in Python, respectively. We statistically tested the differences in microbial compositions by linear discriminant analysis effect size (LEfSe)^28^ using an LDA score cutoff of − 3 and 3.

## Results

### Physical and chemical properties of biochar produced from pyrolysis of water hyacinth

Here we aimed to study the physical and chemical properties of biochar produced from the pyrolysis of water hyacinth. We obtained biochar weighing about 6 kg from our pyrolysis method. This decrease in mass was consistent with the pyrolysis methods of Masto et al.^16^. The pH, EC, and CEC of the biochar were 7.62, 3.52 ds cm^−1^, and 21.95–14.23 cmol kg^−1^, respectively. Our result was consistent with the earlier research of Zhang et al.^20^, who reported the pH and EC of biochar obtained from pyrolysis of water hyacinth at temperature 500 ℃ ± 50 ℃ at 10.49–10.46 and 21.95–14.23 cmol kg^−1^, respectively. Our biochar had a particle size of less than 0.075 mm, as reported in Table 1.

**Table 1:** Physical and chemical properties of biochar derived from water hyacinth.

	Measured value
pH	7.62
EC	3.516 ds cm^−1^
CEC	21.95–14.23 cmol kg^−1^
Amount of ash	39.07–43.04%
Biochar particle size	Less than 0.075 mm

### Chemical properties analysis of Rangsit soil series

Here we aimed to determine the chemical properties of the Rangsit soil series. We found the pH of the soil samples collected from Klong 4, 5, and 6 to be 4.7, 4.8, and 4.7, respectively (Table 2). This indicated that the Rangsit soil samples had high acidity, and we categorized the soil as clay soil or heavy clay soil according to USDA^29^. The EC of soil from Klong 4, 5, and 6 were 0.94, 0.38, and 1 ms cm^−1^, respectively (Table 2). The soil from Klong 6 had the highest CEC, at 31.58 cmol kg^−1^, while the soil from Klong 5 had the lowest CEC, at 25.10 cmol kg^−1^ (Table 2).

**Table 2:** Chemical properties and organic matter content of soil sample collected from Klong 4, 5, and 6 of Rangsit, Pathum-Thani district, Thailand.

	pH	EC (ms cm^−1^)	CEC (cmol kg^−1^)	OM (%)	P (mg kg^−1^)	K (mg kg^−1^)	Mg (mg kg^−1^)	Ca (mg kg^−1^)
Klong 4	4.7	0.936	28.43	2.71	29	174	3905	925
Klong 5	4.8	0.377	25.10	0.50	18	222	3054	781
Klong 6	4.7	0.997	31.58	4.70	11	252	3576	741

The soil from Klong 4 had the highest OM at 4.7%, whereas the soil from Klong 5 has the lowest OM at 0.50%. Moreover, we found that the soil from Klong 4 contained the highest amount of P, Mg, and Ca at for 29, 3,905, and 925 mg kg^−1^, respectively, while the soil from Klong6 contained the highest amount of Mg at 252 mg kg^−1^ (Table 2).

### Determination of the appropriate ratio between biochar and soil for cultivation

Compared to that of the control group, we found that the soil mixture comprising 1:10 of biochar to soil sample caused the greatest increase in pH level in every soil sample. The average pH level of the mixture significantly increased to 6.82, while the control group had the lowest average pH of 5.08 (P < 0.05) (Table 3). Moreover, compared to the control group, we found that the mixture comprising 1:10 of biochar to soil sample also caused the greatest increase in EC level in every soil sample. The average EC of the soil mixture significantly increased to 3.55 ms cm^−1^, whereas the control group had the lowest average EC of 0.42 ms cm^−1^ (P < 0.05). Therefore, we concluded that the appropriate ratio of biochar to soil sample is 1:10.

**Table 3:** Average of pH and EC of the soil mixtures containing different ratios of biochar to soil sample collected from Klong 4, 5, and 6 (different lowercase letters in the column indicate significant differences at the P < 0.05 level following two-way ANOVA analysis and DMRT).

Ratio between biochar and soil sample	Average	
	pH	EC (ms cm^−1^)
0:10 (control)	5.08^d^	0.42^d^
0.25:10	6.35^c^	1.19^c^
0.5:10	6.63^b^	2.16^b^
1:10	6.82^a^	3.55^a^

### Evaluation of the growth of water convolvulus and analysis of chemical properties of the soil mixtures after cultivation

We evaluated the growth of the water convolvulus after cultivating the plant in the soil mixtures described above for 23 days by measuring the shoot height and weight of the plant. To determine the chemical properties of the soil mixtures after cultivation, we also measured the pH and EC of the soils.

Comparing the average height of the plants grown in the soil mixtures comprising different proportions of biochar, soil, and sand, we found that plants grown in different soil mixtures were significantly different in height (P < 0.05). The average height of plants grown in the soil mixture containing 50 of biochar, 400 g of soil, and 100 g of sand was the greatest, at 20.45 cm, while that of the plants grown in the control soil mixture containing 0 g of biochar, 400 g of soil, and100 g of sand was the lowest, at 16.30 cm (P < 0.05) (Table 4 and Supplementary Fig. 2).

**Table 4:** Average shoot height and weight of plants grown in soil mixtures containing different proportions of biochar, soil collected from Klong 4, 5, and 6, and sand.

Proportion of biochar, soil, and sand	Average		
	Height (centimeters)	Weight (grams)	
		With root	Without root
0:400:100	16.30^c^	1.53^b^	1.36^c^
25:400:100	17.00^b^	2.16^a^	1.75^b^
50:400:100	20.45^a^	2.23^a^	2.52^a^

We performed the measurement after 23 days of cultivation (different lowercase letters in the column indicate significant differences at the P < 0.05 level following two-way ANOVA analysis and DMR test).

The average plant weight with root was not significantly different between the plants grown in the soil mixture comprising 25 g of biochar, 400 g of soil, and 100 g of sand, and that comprising 50 g of biochar, 400 g of soil, and 100 g of sand (P > 0.05). The average weight of the plants with roots grown in the soil mixture comprising 50 g of biochar, 400 g of soil, and 100 g of sand was the greatest, at 2.23 g, while that of the plants grown in the control soil mixture comprising 0 g of biochar, 400 g of soil, and 100 g of sand was the lowest, at 1.53 g (P > 0.05) (Table 4).

The average plant weight without root was significantly different among the plants grown in the soil mixtures comprising different proportion of biochar, soil, and sand (P < 0.05). The average weight of plants grown in the soil mixture comprising 50 g of biochar, 400 g of soil, and 100 g of sand was the greatest, at 2.52 g, while that of the plants grown in the control soil mixture comprising 0 g of biochar, 400 g of soil, and 100 g of sand was the lowest, at 1.36 g (P < 0.05) (Table 4).

When we determined the average pH of the soil mixtures containing different proportions of biochar, soil collected from Klong 4, Kong 5, and Klong 6, and sand after the cultivation of water convolvulus, we found no significant differences in the average pH of the soil mixtures (P > 0.05). The average pH of the soil mixture comprising 0 g of biochar, 400 g of soil, and 100 g of sand was 7.56. That of the soil mixture comprising 25 g of biochar, 400 g of soil, and 100 g of sand was 7.53, and that of the soil mixture comprising 50 g of biochar, 400 g of soil, and 100 g of sand was 7.57 (P > 0.05) (Table 5). The average EC was not significantly different among the soil mixtures (P > 0.05). The average EC of the soil mixture comprising 0 g of biochar, 400 g of soil, and 100 g of sand was 0.70 ms cm^−1^. That of the soil mixture comprising 25 g of biochar, 400 g of soil, and 100 g of sand was 0.69 ms cm^−1^, and that of the soil mixture comprising 50 g of biochar, 400 g of soil, and 100 g of sand was 0.68 ms cm^−1^ (P > 0.05) (Table 5).

**Table 5:** Average pH and EC of plants grown in the soil mixtures comprising different proportions of biochar, soil collected from Klong 4, 5, and 6, and sand.

Proportion of biochar, soil, and sand	Average	
	pH	EC (ms cm^−1^)
0:400:100	7.56^a^	0.70^b^
25:400:100	7.53^a^	0.69^b^
50:400:100	7.57^a^	0.68^b^

We performed the measurement after 23 days of cultivation (different lowercase letters in the column indicate significant differences at the P < 0.05 level following two-way ANOVA analysis and DMR test).

We collected two groups of soil after cultivation for 16S amplicon sequencing. The first group of soils, containing samples in triplicate (C1–C3), was from the soil control without biochar amendment (0 g of biochar, 400 g of soil, and 100 g of sand). Another group of soils containing samples in triplicate (B1–B3) was from the soil treated with the proportion of biochar that promoted the optimal growth of water convolvulus (50 g of biochar, 400 g of soil, and100 g of sand).

### Effect of biochar on soil bacterial community

In Supplementary Table 1 we summarize the Illumina sequencing PE reads of 6 soil samples. In total, we obtained 321,898 raw reads from the machine. Individual sequencing samples contained 18,904–83,216 reads, with the lowest number in B3 and the highest in B2. The average raw read in each sample was 53,650 reads per sample. After sequencing QC and host-removing steps, the remaining sequences varied from 95.18 to 97.19%.

**Figure 1:**
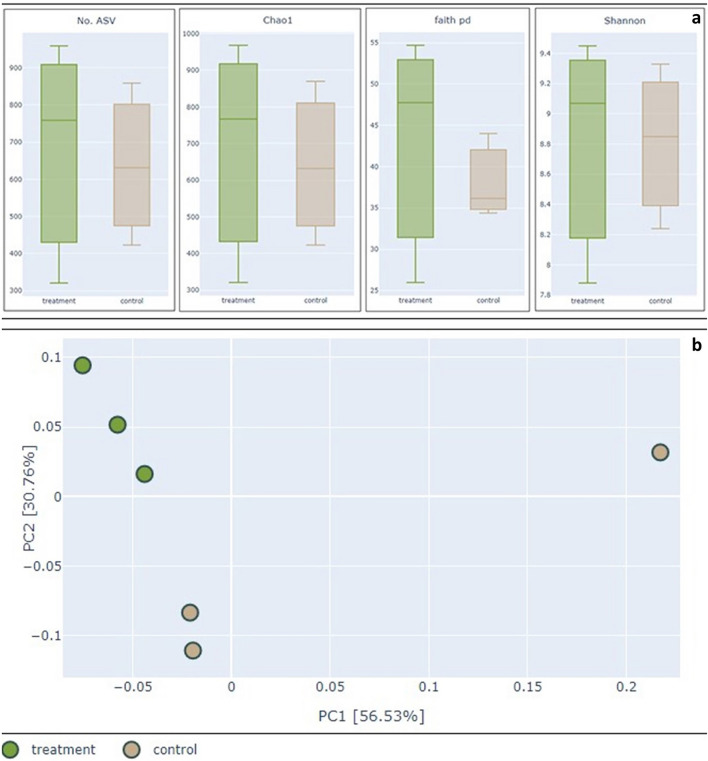
(**a**) Boxplots of alpha diversity index including number of ASVs, Chao1 richness estimator, Faith’s phylogenetic diversity, and Shannon diversity index. (**b**) Principal coordinate analysis (PCoA) from the Bray–Curtis distant matrix.

We studied the microbial community by clustering individual sequences to the ASVs and calculating the diversity index. The result from ASV construction showed that the numbers of ASVs varied threefold in the soil samples, ranging from 321 to 959 ASVs, with the lowest number in B3 and the highest in B2. The White's non-parametric t-test of alpha diversity index (Chao1, Faith’s PD, and Shannon) between control soil and biochar soil was not significantly different (P-value > 0.05) (Fig. 1a). We measured the dissimilarity of bacterial communities between control soil and biochar soil using the Bray–Curtis method. The PCoA of the Bray–Curtis distant metric (Fig. 1b) shows that all soil samples with added biochar (quadrant 4) clustered separately from the control soil samples (quadrant 3). However, one control soil sample (C2) was separate in quadrant 2. The PCoA plot would be expected to reflect the different microbial profiles of different soil content. However, the result from permutational multivariate analysis of variance showed no significant difference in microbial profiles between the two types of soil samples.

### Taxonomic classification

**Figure 2:**
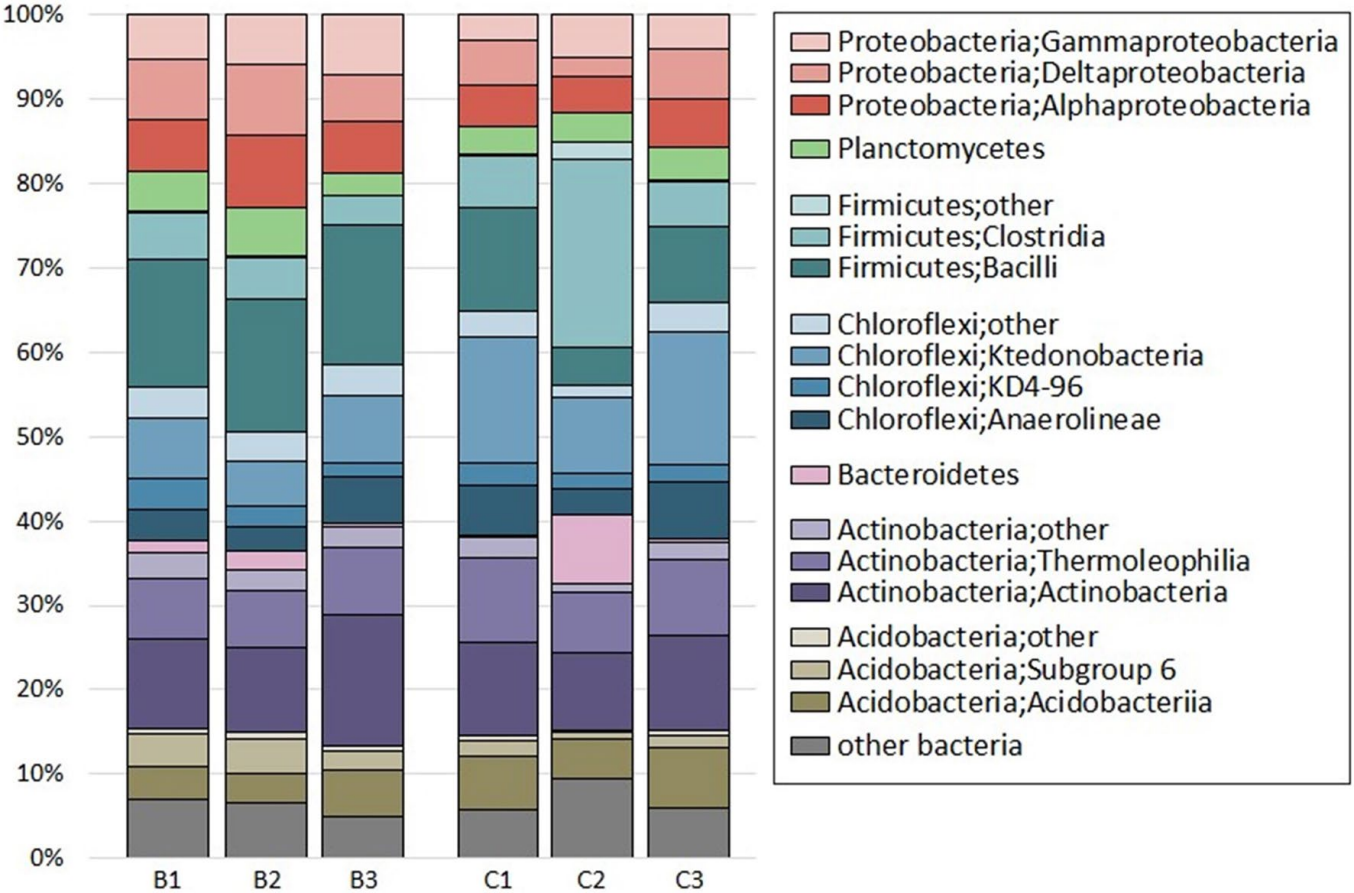
Bacterial class–phyla distribution profiles of microbial diversity between soil with and without biochar (B1–B3 and C1–C3).

Taxonomic classification of the bacterial community from soil samples showed that only 3.12% of all ASVs could not be assigned their taxonomy; most of the remaining sequences could be roughly assigned to phylum levels. For overall bacterial diversity, four major phyla were shared among all samples. *Actinobacteria* were the predominant phylum in all soil, accounting for 21.52% of the total sequences on average, followed by phyla *Firmicutes* and *Chloroflexi* as the second and third most abundant taxa (20.68% and 20.21%, respectively) (Fig. 2). The fourth major phylum was *Proteobacteria*, accounting for 16.76% of all sequences. Other phyla, including *Acidobacteria*, *Planctomycetes*, and *Bacteroidetes*, were found as minorities.

*Actinobacteria* constituted 17.42–25.88% of the total samples. The majority of this phylum belongs to *Actinobacteria* (class) and *Thermoleophilia*, which are accounted for 8.04–11.25%.

In contrast, *Bacilli* (12.27%) and *Clostridia* (7.92%) were the most represented classes in *Firmicutes*.

The *Chloroflexi* were dominated by *Anaerolineae*, KD4-96, and *Ktedonobacteria*. Moreover, *Proteobacteria* were dominated by various bacterial classes including alpha, delta, and gamma *Proteobacteria*, which accounted for 5.99%, 5.75%, and 5.01%, respectively.

**Figure 3:**
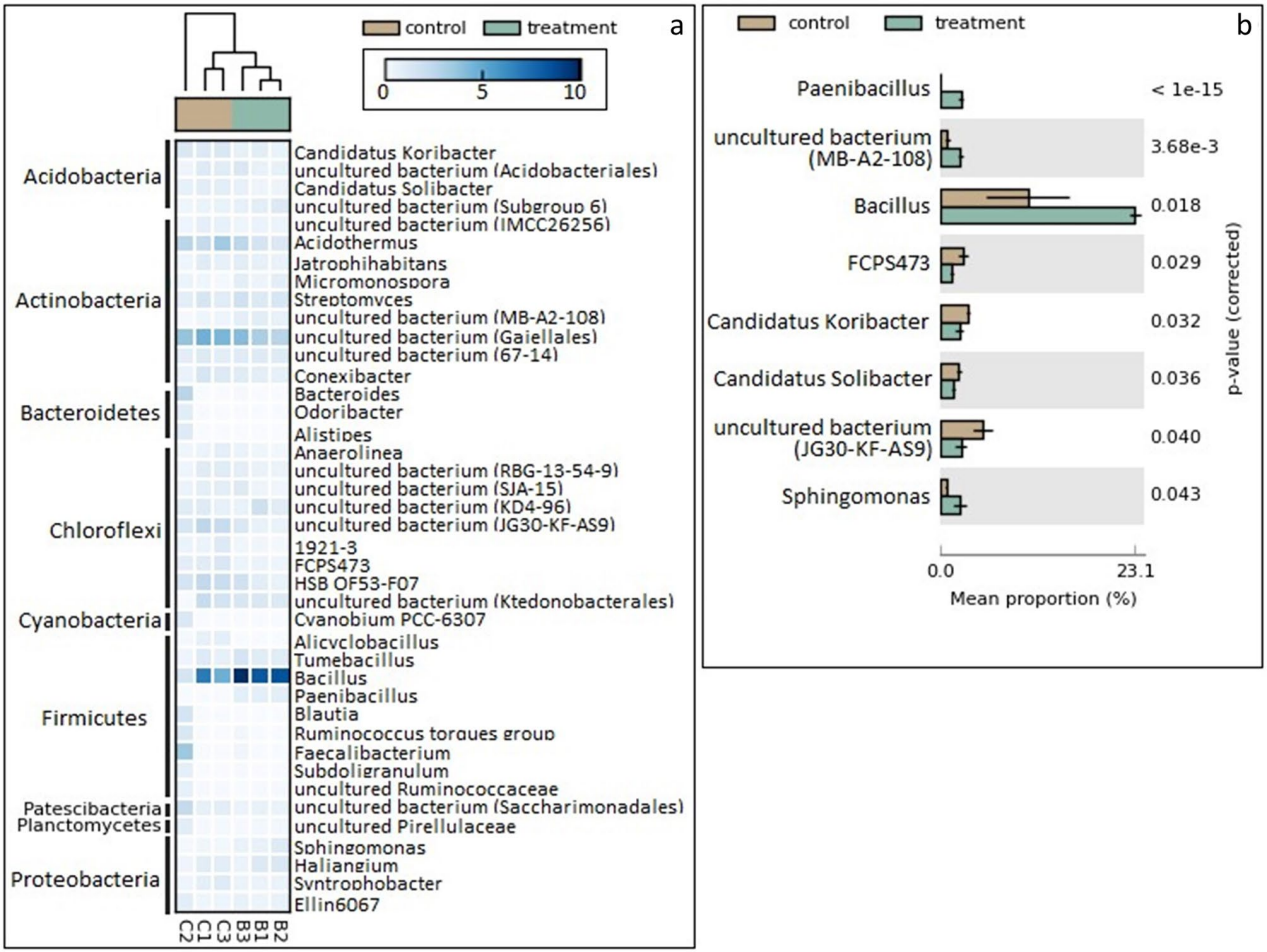
(**a**) Clustered heat map of bacterial profiles at the genus level between soil with and without biochar. (**b**) The difference in the mean proportion of bacterial genus (White's non-parametric t-test; P-value < 0.05).

Clustered heat map of bacterial profiles showing that bacterial phylum *Firmicutes* and genus *Bacillus* and *Paenibacillus* are dominant in the soil community with biochar amendment (treatment), and their expression is more abundant than those in the soil community without biochar amendment (control). Bacteria that are highly abundant in the soil community without biochar amendment are bacterial phylum *Actinobacteria*, genus *Acidothermus*, and bacterial order *Gaiellales* (uncultured bacterium) (Fig. 3a). For the mean proportion, bacterial genus *Bacillus* dominated in the soil community with biochar amendment, followed by *Paenibacillus* and *Shingomonas*. For the soil community without biochar amendment, an uncultured bacterium (JG30-KF-AS9) was the most dominant, followed by genus *Candidatus* (uncultured bacterium) (Fig. 3b).

**Figure 4:**
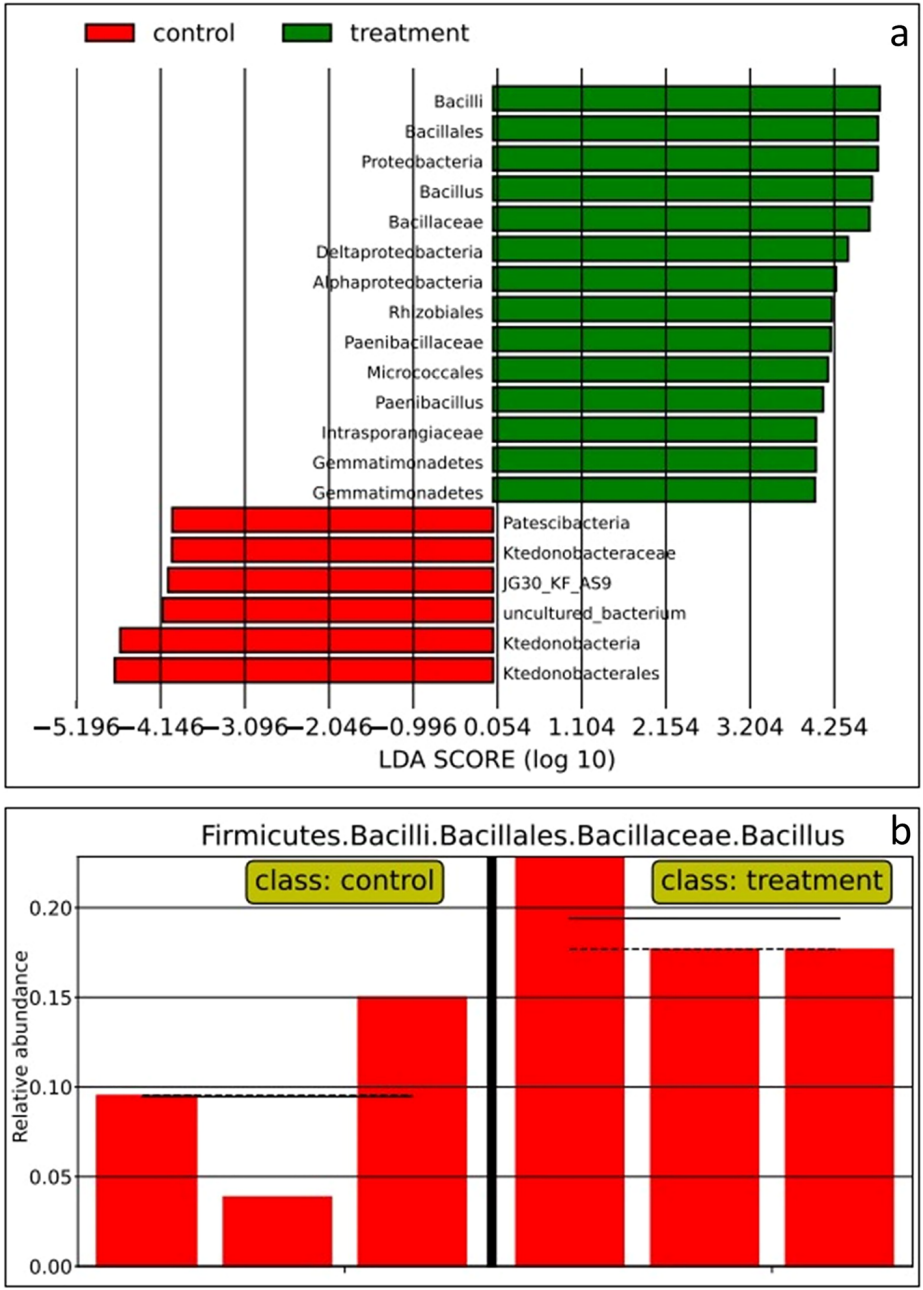
(**a**) Histogram of LDA score of microorganism biomarker computed by LEfSe comparing soil community with and without biochar amendment. (**b**) Relative abundance of Bacillus in biochar soil amendment community (right) and that of Bacillus in non-biochar amendment community (left).

After using LEfSe^28^, we found bacteria that were dominant in the soil community with biochar amendment. The software detected 14 bacterial taxa showing statistical and biological differences in the soil community with biochar amendment, with LDA score above 3 (Fig. 4a). The most dominant bacteria in the soil community with biochar amendment belonged to *Bacilli* with a high LDA score of more than 4.254 orders of magnitude. The second most dominant bacteria in the soil community with the biochar amendment belonged to *Bacillales*, followed by *Proteobacteria*, *Bacillus*, *Bacillaceae*, *Deltaproteobacteria, Alphaproteobacteria*, *Rhizobiales, Paenibacillaceae*, *Micrococcales*, *Paenibacillus*, *Intrasporangiaceae*, and *Gemmatimonadetes*. For the soil without biochar amendment, the software detected 6 bacterial taxa showing statistical and biological differences in the soil community without biochar amendment, with an LDA score above 3. The most dominant bacteria in the soil community without biochar amendment belonged to *Ktedonobacterales*, with a high LDA score of more than 4.146 orders of magnitude. The second most dominant bacteria in the soil community without biochar amendment belonged to *Ktedonobacteria*, followed by the uncultured_bacterium JG30_KF_AS9, *Ktedonobacteraceae*, and *Patescibacteria*. Moreover, we identified bacteria that exhibited high relative abundance in the biochar soil amendment community compared with the non-biochar soil amendment community. We found that *Bacillus* was highly abundant in the amended soil community and its expression was twice that in the non-amended soil community (Fig. 4b). We also identified bacteria that exhibited a high relative abundance in the non-biochar soil amendment community compared with the biochar soil amendment community. We found the expression of *Ktedonobacterales* in the non-biochar amendment community to be 3 times greater than that in the biochar-amended soil community.

## Discussion

Here we reported the utilization of biochar derived from water hyacinth (*Eichornia crassipes*) for acid soil amendment. The appropriate amount of biochar in soil mixture has a positive effect on both acid soil properties and the microbiome of the soil, thus promoting the growth of water convolvulus.

In our study we demonstrated that biochar can significantly increase the pH and EC of our acid soil samples collected from Klong 4, Klong 5, and Klong 6. When the pH and EC of the soil were higher, the plant growth increased. We found that 50 g of biochar, 400 g of soil, and 100 g of sand caused the highest increase in pH level from 4.73 to 6.82 and it was the optimum condition for growth of water convolvulus.

When the soil pH is lower than 5, Al^3+^ in the soil is dissolved^30^. Since Al^3+^ targets the root tips, it causes inhibition of cell elongation and cell inhibition, arresting the development of the root plant^30^. When the root fails to develop properly, it reduces water absorption and nutrient uptake^30^. Alkaline oxides, carbonates, and silicates in biochar can precipitate with the toxic Al^3+^, generating Al(OH)_3_ and Al(OH)^-^_4_ which have less toxicity to the plant root^31,32^. Therefore, biochar amendment can increase the absorption of water and nutrients, promoting plant development.

Biochar derived from water hyacinth has a high exchangeable size and porosity^33^. These two characteristics of biochar are beneficial to crop growth since they promote the water retention and nutrient retention capacity of soil, maintaining microorganisms and increasing efficiency of fertilizer usage^16^. The pores can also enhance infiltration of water from ground surface to the topsoil^34^. Application of rice husk biochar can increase the capacity of soil aggregation, thus preventing soil degradation and promoting crop production^35,36^. Nelissen et al. ^37^, demonstrate that biochar can improve soil physical properties by decreasing bulk density from 1.47 to 1.44 mg m^−3^. Biochar has demonstrated its ability to improve the physical properties of soil—such as water holding capacity, retention of nutrients, stability of soil aggregation, and bulk density. These characteristics can greatly benefit soil fertility^38^.

The results shown in Figs. 3a, b, and 4a proved that treating soil with biochar—changing the pH of the soil– can alter microbial diversity in soil. Our results were consistent with the work of Wang et al.^39^, demonstrating that pH has a greater influence on species richness of bacterial communities than nutrient. *Bacillus* was most dominant and most highly abundant in the soil community with biochar amendment; its expression was twice as high as that in the soil community without biochar amendment. This result indicates that *Bacillus* developed optimally when the acidity of the soil was decreased and when the amount of the *Bacillus* increased, it promoted the growth of water convolvulus. *Paenibacillus* and *Sphingomonas* were also dominant in the soil with biochar amendment. *Bacillus*, *Paenibacillus*, and *Sphingomonas* were identified as plant-growth-promoting rhizobacteria. This type of bacteria has been used to promote environmentally friendly plant growth; therefore, it has been used instead of chemical fertilizer or pesticides^40^. The bacteria inhabit the rhizosphere—the location where direct and indirect mechanisms beneficial to the plant occur, including nutrient uptake^40,41^. Direct mechanisms include producing phytohormones such as indole-3-acetic acid (IAA) in the form that is ready to be taken up by roots to promote plant development, solubilization of phosphorus, and nitrogen fixation^40–45^. Indirect mechanisms include preventing phytopathogen invasion by induction of plant systematic resistance and maintenance of soil aggregation^40,42^. For the non-biochar amendment community, bacteria genus *Candidatus* was dominant in the control group, with a pH of 4.75. Our result was consistent with the study of Willims et al.^46^, demonstrating that *Candidatus udaeobacter* showed the highest abundance in acid soil with a pH ranging from 4.7 to 5.2. *Solibacter usitatus* was reported to produce biofilm supporting the survival of the microorganism under harsh conditions such as an acidic water and soil system^47,48^. Bacterial order *Gaiellales* are highly abundant in the soil community without biochar amendment. *Gaiellales* is reported to thrive more vigorously in soil under acidic condition^49^.

## Conclusions

We first reported the utilization of biochar derived from water hyacinth (*Eichornia crassipes*) for acid soil amendment. We found that 50 g of biochar, 400 g of soil, and 100 g of sand caused the highest increase in pH level and this mixture provided the optimum conditions for growth of water convolvulus. This can be used for future researches to use our condition of biochar mixture for acid soil alleviation and promote optimum growth of the plant.

We also demonstrated that biochar can alter the microbial diversity in soil by changing the pH in soil. *Bacillus* was the most dominant and highly abundant in the soil community with biochar amendment, indicating that *Bacillus* developed optimally when the acidity of the soil was decreased and when the amount of the *Bacillus* increased, it promoted the growth of water convolvulus. Our results show that biochar derived from water hyacinth can be used for acid soil amendment and modify the microbial community beneficially to promote plant development.

## Supplementary Information

Supplementary Table S1.

Supplementary Figure 1.

Supplementary Figure 2.

## Data Availability

The data that support the findings of this study are available from the corresponding author upon reasonable request.
