# Supplementary material for: Alleviation of soil acidification and modification of soil bacterial community by biochar derived from water hyacinth Eichhornia crassipes
Source: Sci Rep. 2023 Jan 9;13:397. doi: 10.1038/s41598-023-27557-9 (PMC9829722; doi:10.1038/s41598-023-27557-9)
Supplement: Supplementary file 1 — Supplementary Table S1. [file 41598_2023_27557_MOESM1_ESM.docx]

**Supplementary Table 1** Summary of sequencing data and diversity index of the 16S rRNA gene sequences.

| **Group** | **Sample Id** | **No. sequences** | | **Alpha diversity index** | | | |
| --- | --- | --- | --- | --- | --- | --- | --- |
|  |  | **raw reads** | **cleaned reads** | **#ASV** | **chao1** | **ace** | **Shannon** |
| Treatment | B1 | 60,111 | 57,527 | 759 | 767 | 767 | 9.1 |
|  | B2 | 83,216 | 80,085 | 959 | 968 | 968 | 9.4 |
|  | B3 | 18,904 | 18,261 | 321 | 321 | 321 | 7.9 |
| Control | C1 | 53,599 | 51,464 | 631 | 632 | 632 | 8.8 |
|  | C2 | 25,258 | 24,548 | 423 | 423 | 423 | 8.2 |
|  | C3 | 80,810 | 76,917 | 859 | 870 | 870 | 9.3 |
