# Supplementary material for: Alleviation of soil acidification and modification of soil bacterial community by biochar derived from water hyacinth Eichhornia crassipes
Source: Sci Rep. 2023 Jan 9;13:397. doi: 10.1038/s41598-023-27557-9 (PMC9829722; doi:10.1038/s41598-023-27557-9)
Supplement: Supplementary file 2 — Supplementary Figure 1. [file 41598_2023_27557_MOESM2_ESM.docx]

**
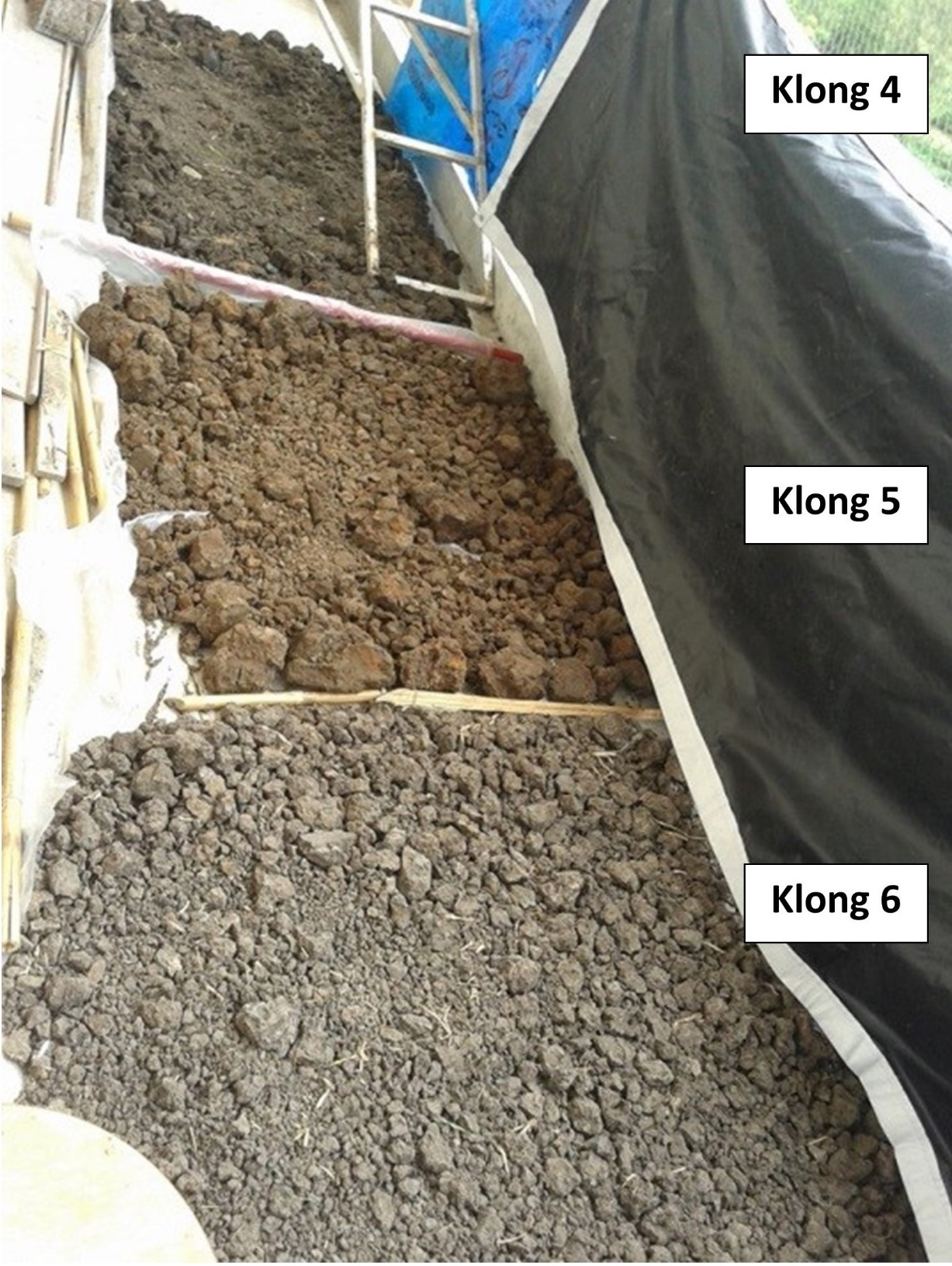
**

**Supplementary Fig. 1** Rangsit soil series of Rangsit, Pathum-Thani district, Thailand, collected from Klong 4, Klong 5, and Klong 6 at the location:14°07'33.7"N 100°43'16.8"E
