# Supplementary material for: Alleviation of soil acidification and modification of soil bacterial community by biochar derived from water hyacinth Eichhornia crassipes
Source: Sci Rep. 2023 Jan 9;13:397. doi: 10.1038/s41598-023-27557-9 (PMC9829722; doi:10.1038/s41598-023-27557-9)
Supplement: Supplementary file 3 — Supplementary Figure 2. [file 41598_2023_27557_MOESM3_ESM.docx]

**
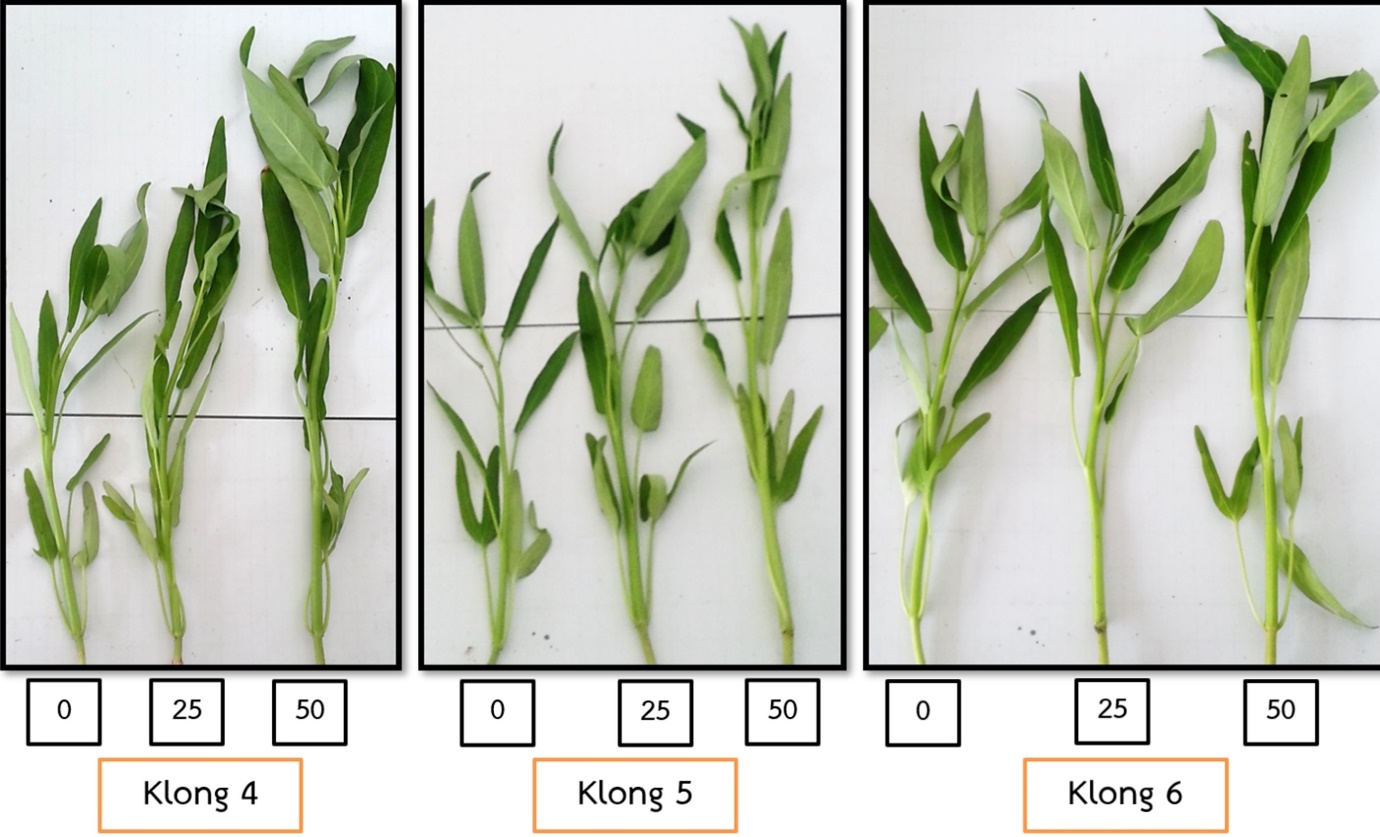
**

**Supplementary Fig. 2** Measurement of shoot height of water convolvulus grown in the soil collected from Klong 4, 5, and 6 mixed with 0, 25, and 50 grams of biochar. We performed the measurement after 23 days of cultivation.
